# Induction of Polyploidy and Metabolic Profiling in the Medicinal Herb *Wedelia chinensis*

**DOI:** 10.3390/plants10061232

**Published:** 2021-06-17

**Authors:** Yung-Ting Tsai, Po-Yen Chen, Kin-Ying To

**Affiliations:** Agricultural Biotechnology Research Center, Academia Sinica, Taipei 115, Taiwan; soloduo@gate.sinica.edu.tw (Y.-T.T.); Chen-Po-Yen@hotmail.com (P.-Y.C.)

**Keywords:** colchicine, flow cytometry, induction of ploidy, medicinal plant, metabolic profiling, polyploid plants, UPLC/MS/MS, *Wedelia chinensis*

## Abstract

*Wedelia chinensis*, which belongs to the Asteraceae family, is a procumbent, perennial herb. It has medicinal anti-inflammatory properties and has been traditionally used as folk medicine in East and South Asia for treating fever, cough and phlegm. In Taiwan, *W. chinensis* is a common ingredient of herbal tea. Previous studies showed that the plant leaves contain four major bioactive compounds, wedelolactone, demethylwedelolactone, luteolin and apigenin, that have potent antihepatoxic activity, and are thus used as major ingredients in phytopharmaceutical formulations. In this study, we set up optimal conditions for induction of ploidy in *W.* *chinensis*. Ploidy can be an effective method of increasing plant biomass and improving medicinal and ornamental characteristics. By using flow cytometry and chicken erythrocyte nuclei as a reference, the DNA content (2C) or genome size of *W. chinensis* was determined to be 4.80 picograms (pg) in this study for the first time. Subsequently, we developed the successful induction of five triploid and three tetraploid plants by using shoot explants treated with different concentrations (0, 0.25, 0.5, 1, 1.5, 2 g/L) of colchicine. No apparent morphological changes were observed between these polyploid plants and the diploid wild-type (WT) plant, except that larger stomata in leaves were found in all polyploid plants as compared to diploid WT. Ultra-performance liquid chromatography coupled with tandem mass spectrometry was used to quantify the four index compounds (wedelolactone, demethylwedelolactone, luteolin, apigenin) in these polyploid plants, and fluctuating patterns were detected. This is the first report regarding polyploidy in the herbal plant *W. chinensis*.

## 1. Introduction

*Wedelia chinensis*, belonging to the Asteraceae family, is a procumbent, perennial herb with bright yellow flowers and a light, camphor-odor. It has medicinal anti-inflammatory properties and has been traditionally used in East and South Asia as a folk medicine for treating fever, cough and phlegm. In addition, *W. chinensis* has also been studied for protection of the liver from toxicity [1,2]. In Taiwan, *W. chinensis* is a common ingredient in herbal tea. Previous studies showed that the plant leaves contain two major bioactive compounds, wedelolactone and demethylwedelolactone, which are responsible for potent antihepatoxic activity, leading to their use as major ingredients in phytopharmaceutical formulations. Four compounds, namely indole-3-carboxyaldehyde, wedelolactone, luteolin and apigenin, isolated from the ethanol fraction of *W. chinensis*, demonstrated potential in prostate cancer prevention and therapy [3,4]. However, these compounds are very rare, making up between 0.162% to 0.390% of air-dried plants [3]. Furthermore, the essential oil of *W. chinensis* showed anti-microbial activity against selected bacterial and fungal strains and anti-inflammation activity against Swiss albino rats [5], and also showed very good antioxidant activity in in vitro and in vivo lung cancer mice [6]. In brief, *W. chinensis* is a valuable medicinal herb.

The artificial induction of polyploidy, containing more than two sets of chromosomes, is a powerful breeding method used in many important crops and ornamentals. Polyploidization can be induced by several antimitotic agents such as colchicine, trifluralin and oryzalin; among them, colchicine is applied in the greatest range of plant species including medicinal plants [7,8,9,10,11,12]. Colchicine is a tricyclic alkaloid extracted from *Colchicine autumnale*, also known as meadow saffron. Colchicine has multiple properties and a complex mechanism of action. It binds to α- and β-tubulin dimers, inhibits microtubule polymerization, and prevents chromosome/chromatid migration during anaphase; as a result, cells contain doubling chromosome numbers [7,13]. The content of secondary metabolites in polyploid genotypes of medicinal plants has been reported to be more than that in the diploid parents [14,15]. For instance, tetraploid *Chamomilla recutita* (a European herbal plant used in aromatherapy and other herbal medicines) accumulated more apigenin than the diploid plant [16]; tetraploid *Artemisia annua* (a traditional Chinese medicinal plant used to treat fever) hairy roots produced up to six times more artemisinin (which has antimalarial activity) than the diploid parent [17]; tetraploid *Tanacetum parthenium* (which has been considered for both medicinal and ornamental uses) showed increased production of parthenloide (a sesquiterpene lactone with antimigraine properties) in the next generation (selfed T_0_ plants) [18]; tetraploid *Echinacea purpurea* (a herb native to North America well known for its effects on immune modulation with wide usage as a dietary supplement, functional food ingredient and food additive) had larger biomass and produced more bioactive compounds (caffeic acid derivatives and alkamines) than the diploid plant [19]. There was around 3-fold higher accumulation of wedelolactone in tetraploid (300.32 μg/g dry weight) than in diploid *Eclipta alba* (93.26 μg/g dry weight); *E. alba* is an annual herbal plant with huge commercialization in hair treatment products in India [20]. Unexpectedly, loss of production or/and new production of secondary metabolites in polyploid plants were also reported [11]. For example, loss of isocarveol geranial and α-bergamotene and a new component of β-bisabolene were detected in colchicine-induced tetraploid plants of *Citrus limon* [21]. The α-terpineol was absent in the essential oil of colchicine-induced tetraploid plants of ajowan medicinal plant, while this compound was present (0.31%) in essential oil in diploid WT [22]. In *Lippia alba*, a major component of citral was not detected in the essential oil of some of the colchicine-induced triploids, and the linalool component was absent from the chemical profile of the majority of tetraploids [23]. New compounds such as α-humulene, α-terpineol and viridiflorol have also been detected in the essential oil of induced tetraploids of *Tetradenia riparia*, these compounds were not seen in the chemical profile of diploid plants [24]. In summary, polyploidy induction may change the expression pattern (including increasing, silencing, and/or up/downregulation) of duplicated genes, affecting the transcription as well as the metabolism, and finally leading to new phenotypic variation [11].

Although there are an abundance of pharmacological data and preclinical studies on *W. chinensis*, information regarding induction of polyploidy in this plant has not hitherto been reported. Plant tissue culture and genetic transformation are efficient tools for the production of phytochemicals and secondary metabolites in a range of medicinal plants [25,26]. In this study, induction of polyploidy from *W. chinensis* is reported. Then, metabolic profiling of four bioactive compounds (i.e., wedelolactone, demethylwedelolactone, luteolin and apigenin) from colchicine-induced polyploidy in *W. chinensis* were further analyzed.

## 2. Results

### 2.1. In Vitro Propagation and Induction of Polyploid Plants

**Figure 1:**
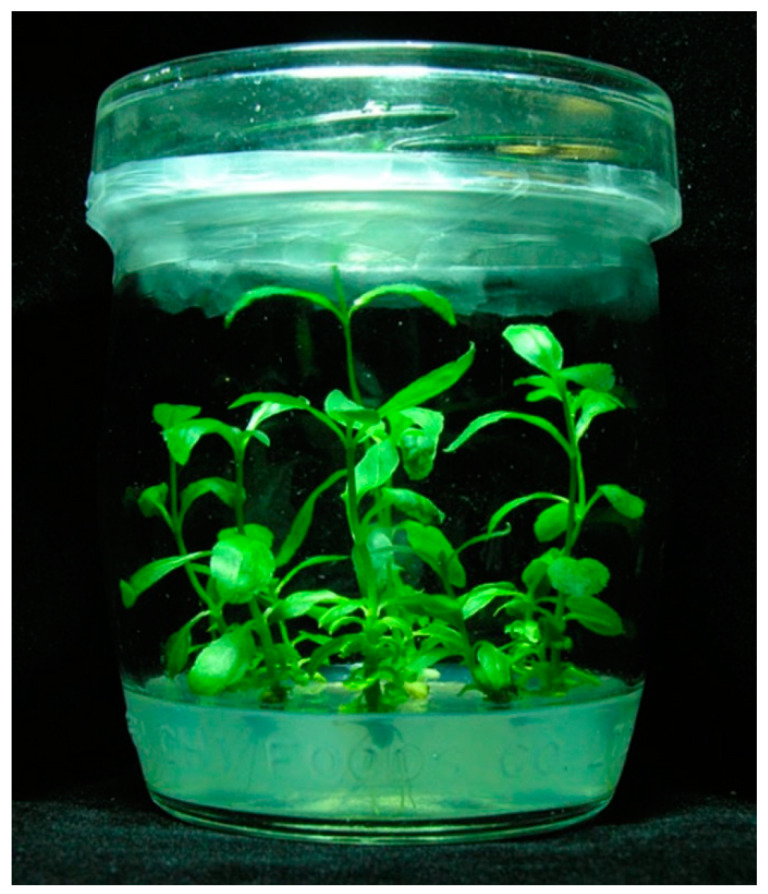
In vitro propagation of *Wedelia chinensis*. Sterile nodal segments were transferred to MS basal medium supplemented with 2% sucrose and grown in a growth chamber for one to two months. Roots were observed at the bottom of the container.

**Figure 2:**
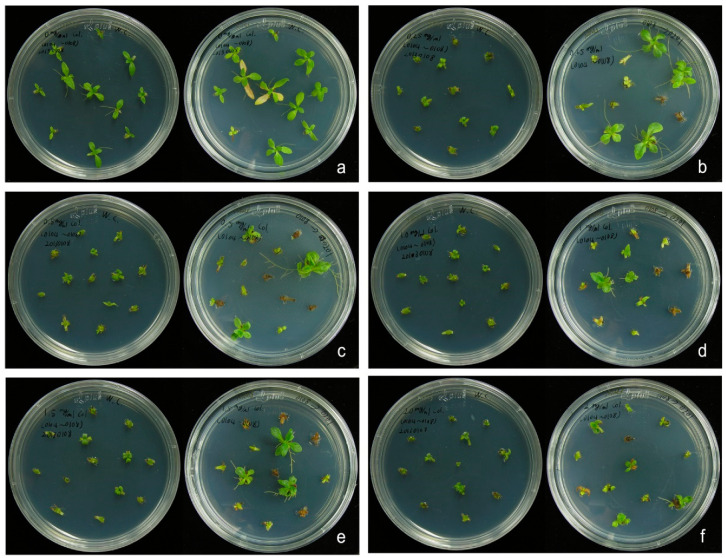
Typical growth patterns of newly regenerated apical shoots which were cultured on MS medium supplemented with different concentrations of colchicine for 4 days and then transferred onto MS medium without adding colchicine for one month. (**a**) No colchicine treatment; (**b**) 0.25 g/L colchicine; (**c**) 0.5 g/L colchicine; (**d**) 1 g/L colchicine; (**e**) 1.5 g/L colchicine; (**f**) 2 g/L colchicine.

**Figure 3:**
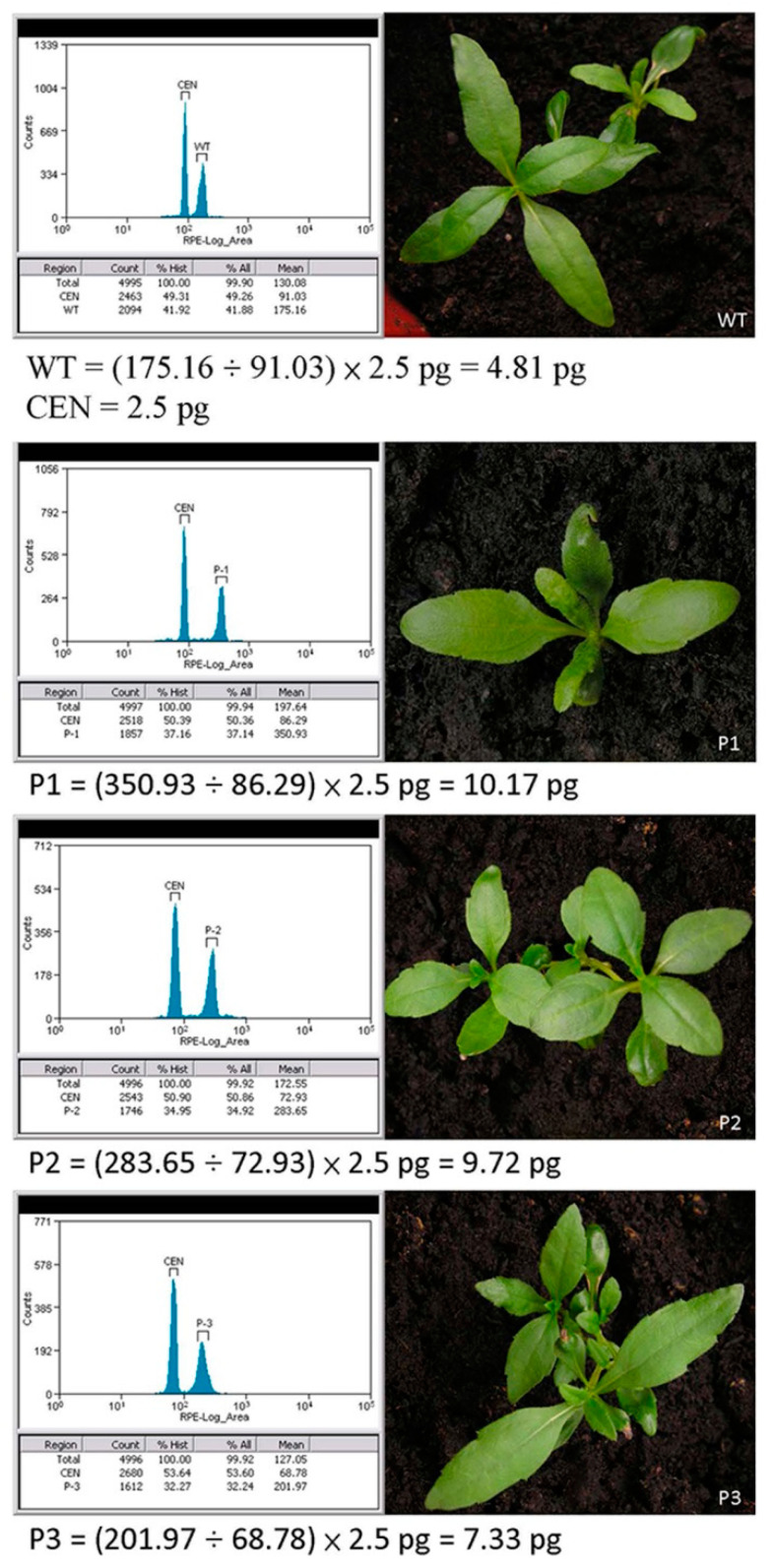
Flow cytometry analysis of selected polyploid plants from *Wedelia chinensis*. Detection of genome size (2C) in diploid (WT), triploid (P3) and tetraploid (P1, P2) plantlets by flow cytometry analysis. CEN represents chicken erythrocyte nuclei. The published genome size of CEN is 2.5 pg.

**Figure 4:**
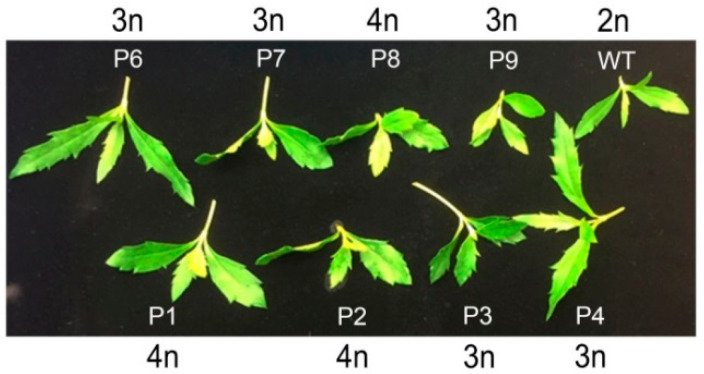
Phenotype of apical shoots from colchicine-induced polyploid plants of *Wedelia chinensis*. Ploidy level in each plantlet is also indicated.

We established in vitro propagation of *W. chinensis* (Figure 1). Then, apical shoots and nodal segments were excised from the in vitro-grown plantlets, and cultured onto MS medium [27] containing different concentrations (0–2 g/L) of colchicine for 4 days. Then, the shoots and segments were transferred onto MS medium supplemented with 0.5 mg/L α-naphthaleneacetic acid (NAA), but not colchicine for 1 month. The newly regenerated apical shoots were cut and transferred onto the above MS medium for another month. As shown in Figure 2a, almost all apical shoots grew healthily on the MS medium without adding colchicine. In contrast, all apical shoots showed growth retardation, and most of them finally died on the MS medium supplemented with 2 g/L colchicine (Figure 2f). Surviving healthy shoots were transferred onto fresh MS medium. Eventually, plantlets with roots were transferred into soil and grown in a greenhouse. To determine the DNA content (2C) or genome size of *W. chinensis*, which has not been reported yet, several top leaves from *W. chinensis* were excised, and the DNA content (2C) of *W. chinensis* was measured by flow cytometry. The DNA content (2C) of *W. chinensis* was determined to be 4.80 ± 0.21 picograms (pg) (mean ± SD), which was derived from five independent experiments (5.15, 4.73, 4.62, 4.68 and 4.81 pg). Next, the DNA content of all colchicine-treated regenerants was determined by comparing with the 2C standard of wild-type (WT) plant. The polyploidy level of each regenerant was obtained by calculating the DNA content of individual regenerants divided by the DNA content of the WT diploid *W. chinensis*. Typical flow cytometry analysis of a few samples is shown in Figure 3. A flow cytometry histogram of a mixture containing a diploid WT and a tetraploid P1 plant is shown in Supplementary Figure S1. Finally, five triploid plants (P3, P4, P6, P7 and P9) and three tetraploid plants (P1, P2 and P8) were obtained in this study (Figure 4). Among these polyploid plants, P1 and P4 were obtained from treatment with 0.5 g/L colchicine, P5 and P7 were obtained from treatment with 1 g/L colchicine, P2 was obtained from treatment with 1.5 g/L colchicine, and P3, P6, P8 and P9 were obtained from treatment with 2 g/L colchicine (Figure 2). P5 was determined to be a triploid plant; however, due to serious infection by microorganisms, P5 finally died.

### 2.2. Characterization of Polyploid Plants

**Figure 5:**
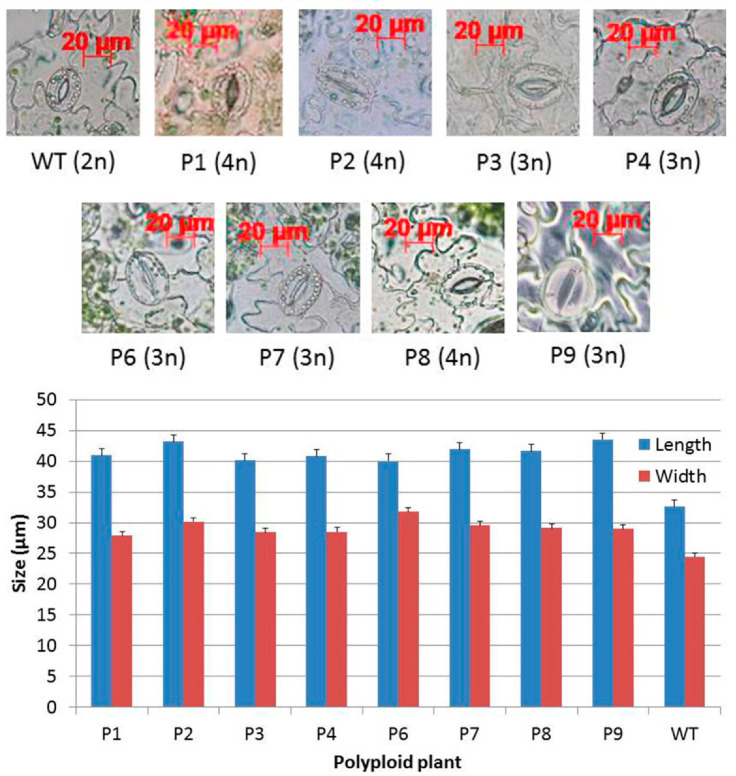
Leaf stomata in diploid wild-type and polyploid plants. Images of leaf stomata under a microscope are shown in the upper panel, and measurement of the size of stomata is shown in the lower panel.

All obtained polyploid plants as well as wild-type plants were grown in a growth chamber. No apparent morphological changes were observed between these polyploid plants and wild-type plants. However, under a microscope, larger stomata were observed in leaves of polyploid plants than in leaves of diploid WT plants (Figure 5).

### 2.3. Identification of Chemical Compounds by UPLC/MS/MS

**Figure 6:**
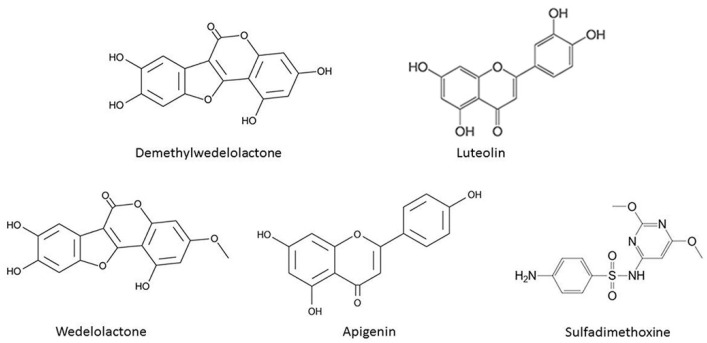
Chemical structure of demethylwedelolactone, luteolin, wedelolactone, apigenin and sulfadimethoxine.

The retention time and information about the molecules (demethylwedelolactone, luteolin, wedelolactone, apigenin) for chemical standards were determined by ultra-performance liquid chromatography coupled with tandem mass spectrometry (UPLC/MS/MS) analysis. The chemical structure of these compounds is shown in Figure 6. Sulfadimethoxine, used as an internal standard, retention time for sulfadimethoxine and 4 standard compounds are shown in Table 1. Data for retention time for luteolin (3.61 min), wedelolactone (3.80 min) and apigenin (4.27 min) matched precisely with a previous report [28]; however, demethylwedelolactone (2.57 min) was not analyzed in the previous study [28]. Furthermore, information about the molecule from precursor ion spectra (*m*/*z*) and product ion spectra (*m*/*z*) for each compound matched perfectly with the existing spectrum data [29,30,31].

### 2.4. Identification and Quantitative Analysis of 4 Metabolites in Wild-Type and Polyploid Plants

To evaluate the content of the 4 metabolites, different tissues (roots, stems and leaves) were independently collected from wild-type *W. chinensis*, and UPLC coupled with tandem mass spectrometry was performed. As shown in Table 2, amounts of demethylwedelolactone, luteolin, wedelolactone and apigenin in leaf tissue were 145.34 μg/g dry weight, 85.85 μg/g dry weight, 18.51 μg/g dry weight, and 34.13 μg/g dry weight, respectively. None of 4 metabolites could be detected in stem tissue; while in root tissue, only very small amounts of luteolin (2.06 μg/g dry weight) were detected, and there was a non-detectable level demethylwedelolactone, wedelolactone and apigenin (Table 2). Thus, we concluded that these 4 metabolites/phytochemicals were mainly found in leaf tissue.

Next, leaves were harvested from polyploid and diploid (WT) plants of *W. chinensis*, and the contents of the 4 metabolites were measured. As shown in Table 3, higher amounts of demethylwedelolactone were detected in polyploid plants P2 (261.67 μg/g dry weight) and P6 (274.65 μg/g dry weight) as compared to diploid WT (183 μg/g dry weight); a similar amount of demethylwedelolactone was detected in polyploid plant P4, and lower amounts of demethylwedelolactone were detected in polyploid plants P1, P3, P7, P8 and P9. With regard to luteolin content, only P4 (107.07 μg/g dry weight) had a similar amount to the WT (104.80 μg/g dry weight), whereas lower amounts of luteolin were detected in the other polyploid plants. With regard to wedelolactone content, only polyploid plant P6 (22.74 μg/g dry weight) had a higher amount than the WT (18.19 μg/g dry weight), whereas lower amounts of wedelolactone were detected in the rest of the polyploid plants. With regard to apigenin content, higher amounts of apigenin were detected in polyploid plants P1 (29.58 μg/g dry weight), P4 (26.03 μg/g dry weight) and P9 (23.50 μg/g dry weight) as compared to WT (21.21 μg/g dry weight); whereas lower amounts of apigenin were detected in the rest of the polyploid plants. In addition, none of the polyploid plants showed higher amounts of all 4 metabolites in any individual polyploid plant as compared to the diploid WT. Typical UPLC profiling of 4 chemical standards and WT is shown in Supplementary Figure S2.

## 3. Discussion

Here, we established optimal conditions for induction of ploidy with colchicine in the medicinal herb *W. chinensis*, and successfully obtained five triploid and three tetraploid plants. To the best of our knowledge, this is the first report of obtaining different levels of ploidy in this plant species. Colchicine is the most commonly used antimitotic agent for induction of polyploidy. Traditionally, 0.005–0.5% colchicine with duration of treatment (from 7 h to 6 days) has been reported to increase genome doubling rate [10]. In this study, five concentrations (0.25, 0.5, 1, 1.5 and 2 g/L, which are equivalent to 0.025, 0.05, 0.1, 0.15 and 0.2%, respectively) of colchicine were used for polyploidy induction from apical shoots or nodal segments of in vitro-grown plantlets. No polyploid plants were obtained at the lowest concentration (0.25 g/L or equivalent to 0.025%) of colchicine; one or two polyploid plants were obtained in samples treated with 0.05%, 0.1% or 0.15% colchicine; and a total of four polyploid plants were obtained from samples treated with 0.2% colchicine. The duration of different concentrations of colchicine in this study was 4 days. The concentration and durations of antimitotic agents (such as colchicine, oryzalin, trifluralin, etc.), different plant organs (such as seeds, apical meristems, flower buds, axillary buds, nodal segments, calli, roots, etc.) and different application methods (such as dipping/soaking, dropping or cotton wool) used for polyploidy induction have been reported elsewhere [8,11].

Polyploidization, no matter whether artificially induced or spontaneously occurring, can cause various phenotypic or metabolic changes such as larger sizes in flowers, fruits, seeds, leaves, roots, biomass, alternation in gene expression or secondary metabolite production [7,10,11,12,32]. However, polyploid plants do not always exhibit higher quality and/or yield in comparison with their diploid parents [7,11]. In this study, no obvious changes except the larger sizes of stomata were seen in polyploid plants in comparison with the diploid WT (Figure 5). Larger stomata in polyploid plants than in diploid parents has been previously reported [11,33,34].

Previous studies showed that at least three bioactive compounds (i.e., wedelolactone, luteolin and apigenin) from herbal extract of the aerial parts of *W. chinensis* synergistically inhibit prostate cancer cell growth in vitro [3,4,28]. Beside these three compounds, demethylwedelolactone (a derivative of wedelolactone) was also monitored in this study. To better understand the tissue distribution of these four secondary metabolites, different tissues including leaves, stems and roots were separately collected from WT *W. chinensis*, and UPLC coupled with tandem MS/MS was quantitatively performed. With the exception of rare amounts (2.06 μg/g dry weight) of luteolin detected in roots, all four metabolites with detectable amounts ranging from 18.51 to 145.34 μg/g dry weight were found majorly in the leaves (Table 2). Unexpectedly, among these four compounds, amounts of demethylwedelolactone (145.34 μg/g dry weight) were much higher than luteolin (85.85 μg/g dry weight), apigenin (34.13 μg/g dry weight) and wedelolactone (18.51 μg/g dry weight) (Table 2). Demethylwedelolactone and wedelolactone are classified as coumestan, and they are structurally similar (Figure 6), and both compounds possess anti-hepatotoxic and anti-cancer properties [35,36,37]. The discovery of more demethylwedelolactone in *W. chinensis* may benefit the medical applications of this herb.

The major purpose of this study was to explore whether production of these four bioactive compounds can be increased in polyploid plants of *W. chinensis*. To our surprise, no individual polyploid plants had higher amounts of all four compounds under examination (Table 3). Instead, significant reduction of all four compounds in 3 (i.e., P3, P7 and P8) out of 8 polyploid plants was detected (Table 3). Specifically, P1 had a higher amount of apigenin as compared to the diploid WT, and lower contents of the other 3 compounds; P2 had higher demethylwedelolactone content and lower contents of the other 3 compounds; P4 had slightly higher demethylwedelolactone, luteolin and apigenin content, and lower wedelolactone content; P6 had higher wedelolactone content and lower content of the other 3 compounds; and P9 had higher apigenin content and lower contents in the other 3 compounds. Most significantly, the tetraploid P2 and the triploid P6 had higher demethylwedelolactone content as compared to diploid WT (Table 3). In brief, fluctuating patterns of increases or decreases in production of the four bioactive compounds in polyploid plants of *W. chinensis* were detected. Since gene expression and regulation are much more complicated in duplicate genomes, the induced polyploids do not always attain the initial presumptions and have higher amounts of active compounds than the diploid parents; and tetraploids, for example, are not twice the size or biomass of diploid parents [7]. Moreover, in orchard grass, transcriptome data of diploid plant after colchicine treatment revealed that extreme decreases in phenylpropanoid, phenylalanine and phytohormone production, and significant increases gene expression are involved in apoptosis and chemical carcinogenesis [10,32]. In this study, two bioactive compounds, namely luteolin and apigenin, are classified as flavone, one class of flavonoids. It is well known that the flavonoid biosynthetic pathway, starting from phenylalanine, leads to production of some classes of flavonoids and anthocyanins [38,39]. Significant decrease in phenylalanine may reduce the downstream products (such as apigenin and luteolin) in flavonoid biosynthesis. Furthermore, production of the secondary metabolites under examination may not all be enhanced in polyploidy. For example, colchicine-induced tetraploids were obtained from a traditional Chinese medicinal plant *Salvia miltiorrhiza*; the amounts of six bioactive compounds (salvianolic B, tanshinone I, tanshinone IIA, dihydrotanshinone I, cryptotanshinone, total tanshinones) were determined by high performance liquid chromatography, and it was found that concentrations of dihydrotanshinone I and total tanshinones in the root extract of the tetraploid plants were significantly higher than diploid WT; however, no differences were detected in the other 4 compounds [34].

In conclusion, we have reported the successful induction, characterization and metabolite profiling of polyploid plants from the medicinal herb *W. chinensis*, and found that the contents of the 4 active compounds under examination in these polyploid plants varied.

## 4. Materials and Methods

### 4.1. Plant Material and Culture Conditions

Although protocols for in vitro propagation from nodal segments or axillary buds of *Wedelia chinensis* have been developed [40,41], we still need to establish an efficient system for propagation and plant regeneration from tissue explants in this species. Fresh whole plant of *W. chinensis* (Osbeck) Merrill was grown in a growth chamber. For in vitro propagation, nodal segments were cut from the fresh plant, sterilized on a 50 mL solution containing 1% sodium hypochloride and three drops of Tween-20 for 20 min, and washed thoroughly with sterile water. They were transferred onto MS basal medium [27] supplemented with 2% sucrose and 0.8% Bacto-agar. The cultures were then incubated in a 22 °C growth chamber under a cycle of 16 h illumination (100 μmol/m^2^/s) and 8 h darkness.

### 4.2. Induction of Polyploidy

Apical shoots and nodal segments were excised from the in vitro-grown plants of *W. chinensis* and considered as explants for polyploidy induction. They were grown in MS medium containing 0, 0.25, 0.5, 1, 1.5 and 2 g/L colchicine for 4 days. Then, the explants were transferred onto basal MS medium without adding colchicine. To initiate root formation, the newly regenerated apical shoots from colchicine-treated explants were cut and cultured on MS medium supplemented with 0.5 mg/L α-naphthaleneacetic acid (NAA), 30 g/L sucrose and 8 g/L agar. Afterwards, the plantlets were transferred onto soil pots and grown in a growth chamber. No phenotypic changes were observed between wild type and colchicine-treated regenerants.

### 4.3. Flow Cytometry Analysis

Isolation and staining plant nuclei were performed by using a flow reagent kit CyStain PI Absolute P (Sysmex Europe GmbH, Norderstedt, Germany). Young leaves were thoroughly chopped using a razor blade in the presence of 1 mL extraction buffer (200 mM Tris, 4 mM MgCl_2_·6H_2_O, 0.5% Triton X-100, pH 7.5) and then filtered through a 50 μm disposable filter. For staining of nuclei, 50 μL propidium iodide staining solution and 5 μL RNase A were added to the tube and mixed well. The tube was incubated on ice for 1 h. The nucleus solution of each sample was analyzed for ploidy level by a flow cytometer (CytoFLEX S Analyzer, Beckman Coulter, Brea, CA, USA). Chicken erythrocyte nuclei (CEN) (BioSure, Grass Valley, CA, USA) were used as an internal biological reference in flow cytometry. The published genome size of CEN is 2.5 picograms (pg) (1 pg = 10^−12^ g) [42]. The diploid genome size of *W. chinensis* was determined to be 4.5 pg, as revealed by flow cytometry analysis.

### 4.4. Measurement of Stomata

To examine stomatal sizes in our polyploid and WT plants, mature leaves were taken from plants which were grown in a green house. The leaf epidermal layer was peeled and then observed with a light microscope (Zeiss Lightsheet Z.1, Jena, Germany) and software (Axio Vision Rel. 4.8, Carl Zeiss Microscopy, Jena, Germany). To examine the length and width of stomata, approximately 50 random-selected stomata were used.

### 4.5. Sample Preparation for UPLC Analysis

Plant extracts were prepared as previously described [3,4] with modifications. Polyploid and wild-type plants were grown in a walk-in growth chamber for 2 months, different tissues were harvested, completely dried in a freeze dryer (FD 12-24P-L-80) for 24 h, and then ground into powder by using a grinder (Retsch Mixers Mill, MM400). In a microfuge tube containing 100 mg sample, 1 mL of 100% methanol was added into the tube. After shocking for 10 min at room temperature, the extraction was centrifuged (13,000 rpm) for 10 min, 300 μL of supernatant was transferred into a 1.5-mL microfuge tube, and equal volume (i.e., 300 μL) of dH_2_O was added into the tube. After adding 6 μL of 12N HCl (pH < 2), hydrolysis was carried out in a 80 °C water bath for 1 h. Then, the pH of the sample was adjusted to 7–8 by adding 6N NaOH, centrifuged (13,000 rpm, 10 min), and the supernatant was used for UPLC analysis. Data was expressed as mean ± standard deviation (S.D.) from three independent experiments of four replicates.

For preparation of chemical standard solution, 3.0 mg of demethylwedelolactone (Sigma-Aldrich, St. Louis, MO, USA), and 1.0 mg each of luteolin (Extrasynthese, Genay Cedex, France), wedelolactone (Sigma-Aldrich, St. Louis, MO, USA) and apigenin (Sigma-Aldrich, St. Louis, MO, USA) were separately dissolved in 10 mL methanol, and the resulting concentrations of standard solutions were 300 μg/mL demethylwedelolactone and 100 μg/mL (luteolin, wedelolactone, apigenin). For preparation of internal standard solution, 1.0 mg of sulfadimethoxine (Tokyo Chemical Industry, Tokyo, Japan) was dissolved in 10 mL of methanol, and the resulting concentration of internal standard solution was 100 μg/mL. To draw the calculation curve, a concentration range from 18.75 to 300 μg/mL was obtained by diluting demethylwedelolactone standard solution (300 μg/mL) with methanol. Similarly, a concentration range from 6.25 to 100 μg/mL was obtained by diluting luteolin, wedelolactone or apigenin standard solution (each of 100 μg/mL) with methanol. Then, equal volumes of different concentrations of standard solutions and internal standard sulfadimethoxine (100 μg/mL) were mixed thoroughly, centrifuged in a microfuge for 10 min at 13,000 rpm. The supernatant was transferred to a new microfuge tube, and 10 μL of standard solution at different concentrations was injected into UPLC for calculation curve analysis.

### 4.6. UPLC-DAD-MS/MS Analysis

The Waters UPLC system (Waters ACQUITY UPLC H-class Plus) coupled with a diode array detector (DAD) and a Thermo Finnigan LCQ Advantage ion-trap mass spectrometer (San Jose, CA, USA) was used for analysis at the Metabolomics Core Facility of the Agricultural Biotechnology Research Center, Academia Sinica, Taiwan. The extracts were separated by UPLC with a column (ACQUITY UPLC BEH, 1.7 μm C18, 2.1 mm × 100 mm) at the flow rate of 0.45 mL/min. The mobile phase consisted of water containing 0.1% (*v*/*v*) formic acid (FA) (mobile phase A) and methanol containing 0.1% (*v*/*v*) FA (mobile phase B), and separations were performed using the following gradients: 10% B from 0 to 0.5 min, 29% B from 0.5 to 4.5 min, 35% B from 4.5 to 8.5 min, 45% B from 8.5 to 11.5 min, 49% B from 11.5 to 11.6 min, 100% B from 11.6 to 14 min, 10% B from 14 to 16 min. The sample injection volume was 10 μL. Eluting peaks were monitored by 330 nm. The chemical structures of these four compounds (namely demethylwedelolactone, wedelolactone, luteolin and apigenin) were elucidated by spectroscopic analysis. Electrospray ionization mass spectrometry data were collected with a LCQ Advantage mass spectrometer (Thermo Finnigan, San Jose, CA, USA) and nuclear magnetic resonance spectra were recorded with Advance 500 and 300 MHz FT nuclear magnetic resonance spectrometers (Bruker, Bremen, Germany) at 500 MHz (^1^H) and 75 MHz (^13^C).

## Figures and Tables

**Table 1:** Chemical standards detected by UPLC/MS/MS analysis.

Compounds	Formula	Molecular Weight	Retention Time (min)	Precursor Ion Spectra (*m*/*z*)	Product Ion Spectra (*m*/*z*)	Reference
Sulfadimethoxine	C_12_H_13_N_4_S	309.07	3.55	309	230	[29]
Demethylwedelolactone	C_15_H_8_O_7_	300.22	2.57	299	227	[30]
Luteolin	C_15_H_10_O_6_	285.04	3.61	285	133	[31]
Wedelolactone	C_16_H_10_O_7_	314.25	3.80	313	298	[31]
Apigenin	C_15_H_10_O_5_	269.05	4.27	269	117	[31]

**Table 2:** Quantitative analysis of 4 metabolites (μg/g dry weight) in different tissues from diploid wild-type *Wedelia chinensis*.

Sample	Demethylwedelolactone	Luteolin	Wedelolactone	Apigenin
Root	n.d.	2.06 ± 0.17	n.d.	n.d.
Stem	n.d.	n.d.	n.d.	n.d.
Leaf	145.34 ± 6.95	85.85 ± 5.54	18.51 ± 3.27	34.13 ± 4.98

n.d. = not detectable. Data from three independent experiments are presented as mean ± S.D. (standard deviation).

**Table 3:** Amounts of 4 metabolites (μg/g dry weight) in leaf tissue from diploid WT and polyploid plants.

Plant No.	Polyploidy	Demethylwedelolactone	Luteolin	Wedelolactone	Apigenin
WT	2n	183.00 ± 6.44	104.80 ± 7.37	18.19 ± 8.10	21.21 ± 4.39
P1	4n	16.48 ± 8.10	87.44 ± 11.23	4.55 ± 1.55	29.58 ± 4.96
P2	4n	261.67 ± 15.63	76.86 ± 10.55	12.85 ± 4.79	20.16 ± 4.67
P3	3n	44.24 ± 11.57	65.68 ± 12.12	6.29 ± 1.48	20.09 ± 3.85
P4	3n	189.90 ± 11.54	107.07 ± 10.16	13.24 ± 4.12	26.03 ± 6.61
P6	3n	274.65 ± 13.67	91.61 ± 17.77	22.74 ± 4.19	15.53 ± 3.59
P7	3n	143.83 ± 14.71	90.44 ± 8.31	14.59 ± 2.58	20.57 ± 2.61
P8	4n	15.61 ± 3.72	50.67 ± 6.08	11.50 ± 2.23	17.54 ± 3.24
P9	3n	124.69 ± 6.26	70.38 ± 7.90	16.43 ± 2.64	23.50 ± 4.08

Data from three independent experiments are presented as mean ± S.D.

## Supplementary Materials

The following is available online at https://www.mdpi.com/article/10.3390/plants10061232/s1, Figure S1: Flow cytometry histogram of a mixture containing a diploid wild-type and a tetraploid P1 plants; Figure S2. Typical UPLC profiling of chemical standards (demethylwedelolactone, luteolin, wedelolactone, apigenin) and leaf extract from wild-type (WT) plants.

## References

[1] Lin S.C., Lin C.C., Lin Y.H., Shyuu S.J. (1994). Hepatoprotective effects of Taiwan folk medicine: *Wedelia chinensis* on three hepatotoxin-induced hepatotoxicity. Am. J. Chin. Med..

[2] Krishnaraju A.V., Tayi V.N.R., Sundararaju D., Vanisree M., Tsay H.S., Subbaraju G.V. (2005). Assessment of bioactivity of Indian medicinal plants using brine shrimp (*Artemia salina*) lethality assay. Intl. J. Appl. Sci. Eng..

[3] Lin F.M., Chen L.R., Lin E.H., Ke F.C., Chen H.Y., Tsai M.J., Hsiao P.W. (2007). Compounds from *Wedelia chinensis* synergistically suppress androgen activity and growth in prostate cancer cells. Carcinogenesis.

[4] Tsai C.H., Lin F.M., Yang Y.C., Lee M.T., Cha T.L., Wu G.J., Hsieh S.C., Hsiao P.W. (2009). Herbal extract of *Wedelia chinensis* attenuates androgen receptor activity and orthotopic growth of prostate cancer in nude mice. Clin. Cancer Res..

[5] Manjamalai A., Jiflin G.J., Berlin Grace V.M. (2012). Study on the effect of essential oil of *Wedelia chinensis* (*Osbeck*) against microbes and inflammation. Asian J. Pharm. Clin. Res..

[6] Manjamalai A., Berlin Grace V.M. (2012). Antioxidant activity of essential oils from *Wedelia chinensis* (*Osbeck*) *in vitro* and *in vivo* lung cancer bearing C57BL/6 mice. Asian Pac. J. Cancer Prev..

[7] Sattler M.C., Carvalho C.R., Clarindo W.R. (2016). The polyploidy and its key role in plant breeding. Planta.

[8] Manzoor A., Ahmad T., Bashir M.A., Hafiz A., Silvestri C. (2019). Studies on colchicine induced chromosome doubling for enhancement of quality traits in ornamental plants. Plants.

[9] Niazian M. (2019). Application of genetic and biotechnology for improving medicinal plants. Planta.

[10] Ahmadi B., Ebrahimzadeh H. (2020). In vitro androgenesis: Spontaneous vs. artificial genome doubling and characterization of regenerants. Plant Cell Rep..

[11] Niazian M., Nalousi A.M. (2020). Artificial polyploidy induction for improvement of ornamental and medicinal plants. Plant Cell Tissue Organ Cult..

[12] Touchell D.H., Palmer J.E., Ranney T.G. (2020). *In vitro* ploidy manipulation for crop improvement. Front. Plant Sci..

[13] Karamanou M., Tsoucalas G., Pantos K., Androutsos G. (2018). Isolating colchicine in 19th century: An old drug revisited. Curr. Pharm. Des..

[14] Salma U., Kundu S., Mandal N. (2017). Artifical polyploidy in medicinal plants: Advancement in the last two decades and impending prospects. J. Crop Sci. Biotechnol..

[15] Pradhan S.K., Gupta R.C., Goel R.K. (2018). Differential content of secondary metabolites in diploid and tetraploid cytotypes of *Siegesbeckia orientalis* L.. Nat. Prod. Res..

[16] Švehlíková V., Repčák M. (2000). Variation of apigenin quantity in diploid and tetraploid *Chamomilla recutita* (L.) Rauschert. Plant Biol..

[17] De Jesus-Gonzalez L., Weathers P.J. (2003). Tetraploid *Artemisia annua* hairy roots produce more artemisinin than diploids. Plant Cell Rep..

[18] Majdi M., Karimzadeh G., Malboobi M.A., Omidbaigi R., Mirzaghaderi G. (2010). Induction of tetraploidy to feverfew (*Tanacetum parthenium* Schulz-Bip.): Morphological, physiological, cytological, and phytochemical changes. HortScience.

[19] Xu C.G., Tang T.X., Chen R., Liang C.H., Liu X.Y., Wu C.L., Yang Y.S., Yang D.P., Wu H. (2014). A comparative study of bioactive secondary metabolite production in diploid and tetraploid *Echinacea purpurea* (L.) Moench. Plant Cell Tissue Organ Cult..

[20] Salma U., Kundu S., Harra A.K., Ali M.N., Mandal N. (2018). Augmentation of wedelolactone through in vitro tetraploid induction in *Eclipta alba* (L.) Hassk. Plant Cell Tissue Organ Cult..

[21] Bhuvaneswari G., Thirugnanasampandan R., Gogulramnath M. (2020). Effect of colchicine induced tetraploidy on morphology, cytology, essential oil composition, gene expression and antioxidant activity of *Citrus limon* (L.) Osbeck. Physiol. Mol. Biol. Plant.

[22] Noori S.A.S., Norouzi M., Karimzadeh G., Shirkool K., Niazian M. (2017). Effect of colchicine-induced polyploidy on morphological characteristics and essential oil composition of ajowan (*Trachyspermum ammi* L.). Plant Cell Tissue Organ Cult..

[23] Julião S.A., Ribeiro C.D.V., Lopes J.M.L., Matos E.M.D., Reis A.C., Peixoto P.H.P., Machado M.A., Azevedo A.L.S., Grazul R.M., Campos J.M.S.D. (2020). Induction of synthetic polyploids and assessment of genome stability in *Lippia alba*. Front. Plant Sci..

[24] Hannweg K., Visser G., de Jager K., Bertling I. (2016). In vitro-induced polyploidy and its effect on horticultural characteristics, essential oil composition and bioactivity of *Tetradenia riparia*. S. Afr. J. Bot..

[25] Nalawade S.M., Tsay H.S. (2004). In vitro propagation of some important Chinese medicinal plants and their sustainable usage. In Vitro Cell. Dev. Biol. Plant.

[26] Gómez-Galera S., Pelacho A.M., Gené A., Capell T., Christou P. (2007). The genetic manipulation of medicinal and aromatic plants. Plant Cell Rep..

[27] Murashige T., Skoog F. (1962). A revised medium for rapid growth and bioassays with tobacco tissue cultures. Physiol. Plant..

[28] Tsai C.H., Tzeng S.F., Hsieh S.C., Lin C.Y., Tsai C.J., Chen Y.R., Yang Y.C., Chou Y.W., Lee M.T., Hsiao P.W. (2015). Development of a standardized and effect-optimized herbal extract of *Wedelia chinensis* for prostate cancer. Phytomedicine.

[29] MassBank Europe MassBank Record EA018355. https://www.massbank.eu.

[30] Li M., Si D., Fu Z., Sang M., Zhang Z., Liu E., Yang W., Gao X., Han L. (2019). Enhanced identification of the *in vivo* metabolites of *Ecliptae herba* in rat plasma by integrating untargeted data-dependent MS^2^ and predictive multiple reaction monitoring-information dependent acquisition-enhanced product ion scan. J. Chromatogr. B.

[31] Cheruvu H.S., Yadav N.K., Valicherla G.R., Arya R.K., Hussain Z., Sharma C., Arya K.R., Singh R.K., Datta D., Gayen J.R. (2018). LC-MS/MS method for the simultaneous quantification of luteolin, wedelolactone and apigenin in mice plasma using hansen solubility parameters for liquid-liquid extraction: Application to pharmacokinetics of *Eclipta alba* chloroform fraction. J. Chromatogr. B.

[32] Zhou K., Fleet P., Nevo E., Zhang X., Sun G. (2017). Transcriptome analysis reveals plant response to colchicine treatment during on chromosome doubling. Sci. Rep..

[33] Kaensaksiri T., Soontornchainaksaeng P., Soonthornchareonnon N., Prathanturarug S. (2011). In vitro induction of polyploidy in *Centella asiatica* (L.) Urban. Plant Cell Tissue Organ Cult..

[34] Chen E.G., Tsai K.L., Chung H.H., Chen J.T. (2018). Chromosome doubling-enhanced biomass and dihydrotanshinone I production in *Salvia miltiorrhiza*, a traditional chinese medicinal plant. Molecules.

[35] Zafar R., Sagar B.P.S. (1999). In vitro plant regeneration of *Eclipta alba* and increased production of coumestans. Fitoterapia.

[36] Sagar B.P.S., Panwar R., Goswami A., Kadian K., Tyagi K., Chugh M., Dalal S., Zafar R. (2006). Pharmacokinetic interactions of antihepatotoxic wedelolactone with paracetamol in wistar albino rats. Pharm. Biol..

[37] Lee Y.J., Lin W.L., Chen N.F., Chuang S.K., Tseng T.H. (2012). Demethylwedelolactone derivatives inhibit invasive growth *in vitro* and lung metastasis of MDA-MB-231 breast cancer cells in nude mice. Eur. J. Med. Chem..

[38] Winkel-Shirley B. (2001). Flavonoid biosynthesis: A colorful model for genetics, biochemistry, cell biology, and biotechnology. Plant Physiol..

[39] To K.Y., Wang C.K., Teixeira da Silva J.A. (2006). Molecular breeding of flower color. Floriculture, Ornamental and Plant Biotechnology, Volume I.

[40] Martin Y.P., Beena M.R., Joseph D. (2003). High frequency axillary bud multiplication and ex vitro rooting of *Wedelia chinensis* (Osbeck) Merr.—A medicinal plant. Indian J. Exp. Biol..

[41] Rahman M.M., Bhadra S.K. (2011). Development of protocol for in vitro culture and rapid propagation of *Wedelia chinensis* (Osbeek) Merr. J. Med. Plant Res..

[42] Animal Genome Size Database. http://www.genomesize.com.

