# Supplementary material for: Induction of Polyploidy and Metabolic Profiling in the Medicinal Herb Wedelia chinensis
Source: Plants (Basel). 2021 Jun 17;10(6):1232. doi: 10.3390/plants10061232 (PMC8235177; doi:10.3390/plants10061232)
Supplement: Supplementary file 1 [file plants-10-01232-s001.zip › plants-1249387-supplementary.pdf]

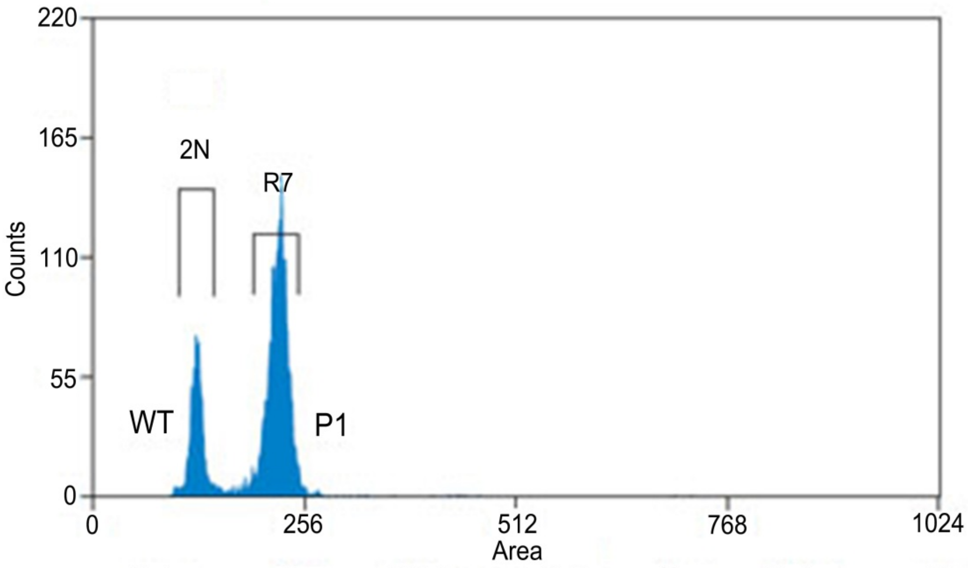

Region	Count	% Hist	% All	Mean	Median	CV
Total	4612	100.00	92.24	199.44	219.00	23.53
2N	1099	23.83	21.96	125.25	125.00	5.65
R7	3249	70.45	64.98	224.14	225.00	4.81

Supplementary Figure S1. Flow cytometry histogram of a mixture containing a wild-type diploid and a tetraploid P1 plants.

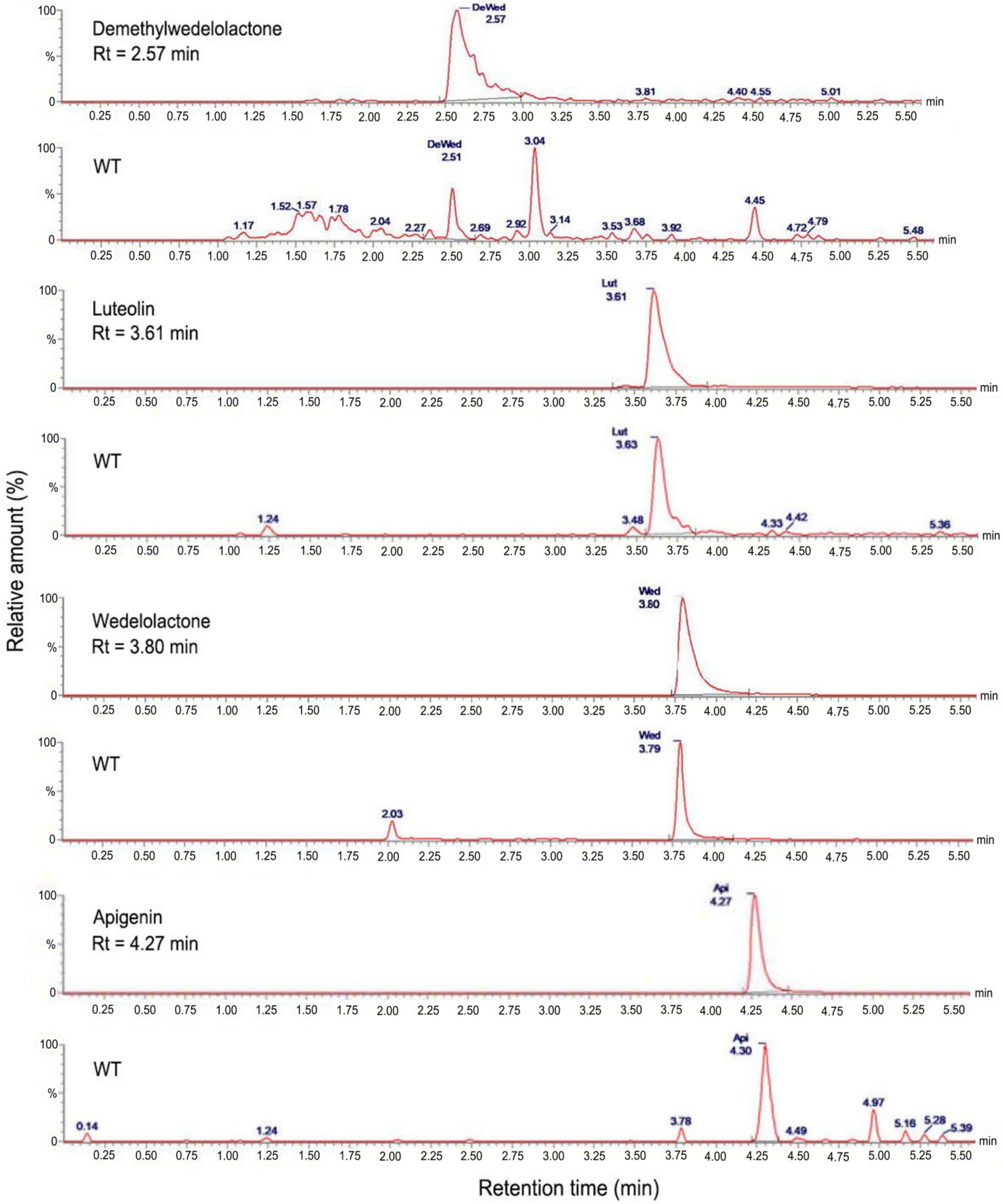

Supplementary Figure S2. Typical UPLC profiling of chemical standards (demethylwedelolactone, luteolin, wedelolactone, apigenin) and leaf extract from wild-type (WT) plants.
